# Innovative Biomaterials for Bone Regrowth

**DOI:** 10.3390/ijms20030618

**Published:** 2019-01-31

**Authors:** Maria Rosa Iaquinta, Elisa Mazzoni, Marco Manfrini, Antonio D’Agostino, Lorenzo Trevisiol, Riccardo Nocini, Leonardo Trombelli, Giovanni Barbanti-Brodano, Fernanda Martini, Mauro Tognon

**Affiliations:** 1 Department of Morphology, Surgery, and Experimental Medicine, University of Ferrara, 44121 Ferrara, Italy; mariarosa.iaquinta@unife.it (M.R.I.); elisa.mazzoni@unife.it (E.M.); marco.manfrini@unife.it (M.M.); 2 Department of Surgery, University of Verona, 37129 Verona, Italy; antonio.dagostino@univr.it (A.D.); lorenzo.trevisiol@univr.it (L.T.); riccardo.nocini@gmail.com (R.N.); 3 Research Centre for the Study of Periodontal and Peri-Implant Diseases, University of Ferrara, 44121 Ferrara, Italy; leonardo.trombelli@unife.it; 4 IRCCS Istituto Ortopedico Rizzoli, 40136 Bologna, Italy; giovanni@barbantibrodano.com

**Keywords:** regenerative medicine, bone, biomaterial, stem cells, growth factors

## Abstract

The regenerative medicine, a new discipline that merges biological sciences and the fundamental of engineering to develop biological substitutes, has greatly benefited from recent advances in the material engineering and the role of stem cells in tissue regeneration. Regenerative medicine strategies, involving the combination of biomaterials/scaffolds, cells, and bioactive agents, have been of great interest especially for the repair of damaged bone and bone regrowth. In the last few years, the life expectancy of our population has progressively increased. Aging has highlighted the need for intervention on human bone with biocompatible materials that show high performance for the regeneration of the bone, efficiently and in a short time. In this review, the different aspects of tissue engineering applied to bone engineering were taken into consideration. The first part of this review introduces the bone cellular biology/molecular genetics. Data on biomaterials, stem cells, and specific growth factors for the bone regrowth are reported in this review.

## 1. Introduction

Bone pathologies are the main causes of disability. With the increase in life expectancy, it is foreseeable that millions of people in many countries will be affected by diseases affecting the bones. The diseases affecting the bone, both acute, such as fractures, and chronic, i.e., osteoporosis and tumors, require treatments which involve the use of cells, growth factors and bone substitutes, as biomaterials/scaffolds, with biocompatibility, osteoinductive, and osteoconductive properties [1,2,3].

Many scientific studies are conducted to identify new biomaterials that promote bone regrowth in a limited time and in a personalized manner. In addition, the scaffold properties can be optimized for different applications in the fields of maxillofacial, odontoiatric, and orthopedic sciences/clinics. The scaffolds used in the clinical practice are of various chemical/physical nature [2], such as ceramics [4], polymers [5] and composite biomaterials [6]. Recent works have hypothesized the use of human mesenchymal stem cells (hMSCs) in association with particular scaffolds in order to guarantee the regeneration of the bone tissue. Several studies report that hMSCs isolated from the bone marrow aspirate [7,8] or adipose tissue in combination with scaffolds [9,10] are able to induce the bone regeneration. The targeted interventions are destined to grow in proportion to the number of patients affected by diseases and disabilities of the skeletal system. 

## 2. Bone Biology

Bone is a highly dynamic connective tissue, which provides adequate mechanical strength and structural support to the body. At macroscopic level, it is classified as cortical or compact bone and cancellous or trabecular [11]. Both these compartments are characterized by an orchestrated 3D architecture with high structural complexity.

Moreover, bone tissue is composed of inorganic and organic phases. The most prevalent component in the inorganic phase is hydroxylapatite (HA; Ca_10_(PO_4_)_6_(OH)_2_) with citrate, carbonate and ions such as F^−^, K^+^, Sr^2+^, Pb^2+^, Zn^2+^, Cu^2+^, and Fe^2+^. Bone organic phase includes type I collagen and non-collagenous proteins, such as osteocalcin, osteonectin, bone sialoproteins, and various proteoglycans that have an important role in the matrix maturation process and may regulate the functional activity of bone cells [12,13]. 

In addition, bone tissue exhibits four types of cells: osteoblasts, osteocytes, bone lining cells, and osteoclasts [14,15]. 

Osteoblasts are largely known as bone-forming cells and their differentiation is mainly controlled by runt-related transcription factor 2 (RUNX2), as well as other transcription factors [16,17,18]. 

Osteocytes, which are long-lived cells within the bone matrix, derive from the osteoblasts when they become embedded within the bone matrix. During this transition, osteoblasts arrest the production of the extracellular matrix and differentiate into osteocytes. Osteocytes are committed, as main activities, to remove damaged organelles and macromolecules using quality-control pathways, such as the autophagy [19]. Osteocytes express protein sclerostin, which prevents Wnt signaling. The expression of this protein can be inhibited by parathyroid hormone signaling to allow Wnt directed bone formation to occur. Moreover, osteocytes can inhibit osteoclastogenesis by secreting the transforming growth factor β (TGF-β). However, upon stimulation osteoblasts and osteocytes induce bone remodeling because they produce osteoclastogenic factors, such as CSF-1 and RANKL, the receptor activator of the NF-κB ligand [16,17,18,20].

Finally, bone lining cells are quiescent osteoblasts that cover the bone surfaces, where bone resorption or bone formation are not request [21].

On the other hand, the differentiation of osteoclasts, bone-resorbing cells, is regulated by specific cytokines [22] that control their formation, maturation and activity. Moreover, they regulate synthesis of matrix enzymes leading to bone resorption. The knowledge of this mechanism leads to the development of therapeutic agents that can arrest osteoclastogenesis reducing bone loss [23].

The dominant pathways that leads to osteoclast formation and activity is represented by tumor necrosis factor ligand superfamily member 11 (RANKL) and macrophage colony stimulating factor, (M-CSF or CSF-1). RANKL is involved in osteoclast differentiation [24] while CSF-1 is required for the proliferation and survival of osteoclast precursor cells [24].

Osteoblasts, osteoclasts, their precursor cells, and associated cells, e.g., endothelial cells and nerve cells, are made up of specialized units called bone multicellular units [25].

The most important function of bone multicellular units in the adult skeleton is to mediate ‘bone remodeling’, which is a mechanism aimed at maintaining skeleton integrity. In this way, old high mineral density bone, which is subjected to a high prevalence of fatigue micro-fractures is removed through repetitive cycles of bone resorption and bone formation [25].

Normal bone remodeling is necessary for fracture healing and bone adaptation to mechanical use, as well as for calcium homeostasis [26]. Conversely, alterations to bone resorption/formation in this process result in several skeletal diseases. For example, osteoporosis is determined by an excessive resorption by osteoclasts without a corresponding amount of new bone formation by osteoblasts [27], while the contrary may result in osteopetrosis [28].

## 3. Bone Fracture and Diseases

Worldwide more than 20 million patients are annually affected by a loss of bone tissue caused by trauma or disease [29]. In the United States alone, over half a million bone defect repairs occur with a cost of over $2.5 billion [30], while the cost in the European Union is estimated at about 40 billion Euro and is set to increase by 25% by 2025 [31,32]. In addition to trauma, bone healing problems are usually associated with several factors, such as age, sex and infection, as demonstrated by diagnoses, such as osteopenia, osteoporosis and severe dental problems related to tooth loss [12]. For instance, osteoporosis has been recognized as one of the most important disease afflicting the global population alongside hypertension and diabetes mellitus [33], whereas its economic impact is similar to the cost of other major diseases, such as strokes, breast cancer or myocardial infarction. It is characterized by an imbalance in the bone remodeling process that leads to a progressive loss in bone mass and, subsequently, an increase in the fracture risk [34]. The quality of life is notably reduced in patients with osteoporotic fractures due to decreased functional mobility and an indirect increase in professional home-care services. Therefore, it is important to limit the consequences of this pathology with adequate and personalized prevention and treatment.

Recent study analyzed the relationship between sarcopenia, a common geriatric syndrome characterized by the progressive decrease of muscle mass, and fragility fracture [35]. Interestingly, Chalhoub et al. [36] reported that the fracture risk in males was significantly increased with both sarcopenia and osteoporosis respect to patients with sarcopenia or osteoporosis alone. This result demonstrates that the interaction between low bone mass and low lean body mass has an influence on bone quality. To date, there is a great interest on the combined effect of sarcopenia and osteoporosis on fracture risk. In this field, new terms such as “sarco-osteopenia” and “sarco-osteoporosis” were coined by Binkley and Buehring to indicate older people affected by sarcopenia and osteoporosis [37]. 

In addition to osteoporosis, another chronic disease affecting bones is cancer. In general, this disease remains the second-most common cause of death worldwide, despite advances in prevention, early detection and treatment protocols [2]. The International Agency for Research on Cancer (Cancer Research UK data) estimated the total number of new cancer cases in 2008 as 12,662,554 (52.26% men), while ∼21 million new cancer cases are expected for 2030 [2]. Unlike both hematological and solid tumors that are usually diagnosed in elderly people [38,39,40,41], osteosarcoma, mainly affects children, adolescents and young adults [42,43]. Indeed, osteosarcoma is the commonest primary tumor in young subjects in the range of 15–19 years old, while it represents over 10% of all solid cancers in adolescents [44]. In addition, osteosarcoma prevalence is increasing 6–8/million/year in young people [45,46,47,48]. 

Current treatment of osteosarcoma includes surgical resection in association with chemotherapy [49]. During the treatment, active agents such as high doses of methotrexate with leucovorin rescue, doxorubicin and cisplatin [50,51] are administered over the course of 30 weeks [52].

In this context, several biomaterials have been tested as local drug-delivery systems to improve bone cancer treatment and patient management [53,54].

## 4. Tissue Engineering: Stem Cells and Biomaterials in Bone Formation

**Figure 1:**
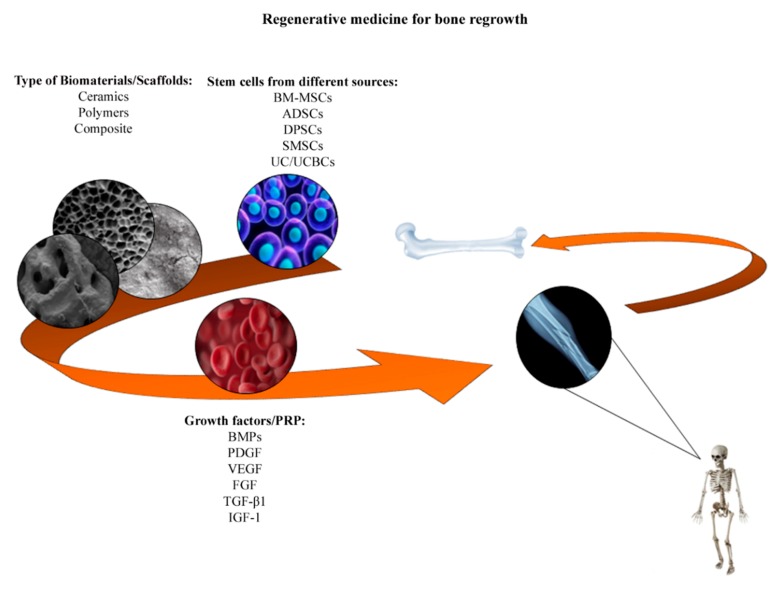
Representation of key elements for bone regrowth. The regenerative medicine improves bone repair using:(i) mesenchymal stem cells (hMSCs) derived from different tissues, including the bone marrow (BM-MSCs), adipose (ADSCs), dental pulp (DPSCs), synovial membrane (SMSCs), umbilical cord (UC) and umbilical cord blood cells (UCBCs); (ii) biomaterials/scaffolds classified in ceramics, polymers and composite; (iii) Platelet rich-plasma (PRP) and growth factors, such as platelet derived growth factor (PDGF), vascular endothelial growth factor (VEGF), fibroblast growth factor (FGF), transforming growth factor-β1 (TGF-β1), insulin-like growth factor 1 (IGF-1) and bone morphogenetic proteins (BMPs).

Bone tissue, in normal conditions, has a particular healing capacity which does not involve scar tissue formation. However, in some cases bone fractures are too complex, for instance, fractures above a critical size lead to non-union fracture end healing failure [55]. Currently, therapeutic strategies based on bone grafting using an auto-graft or an allo-graft show some disadvantages: auto-graft includes implants taken from patient himself/herself and are limited by the bone volume that can be harvested from the iliac crest. This technique also presents surgical risks such as bleeding, inflammation, infection and chronic pain, as well as damage to the donor site and morbidity, deformity, hypersensitivity and scarring. Allografts (implants from a donor) also have some limitations, such as the lack of donors, high costs, the need for sterilization and the risk of infectious agent transmission or immune mediated tissue rejection [1,55,56]. These limitations and disadvantages associated with auto- and allograft approaches indicate a clinical need for alternative therapeutic strategies aimed at bone healing. Thus, tissue engineering has employed new biomaterials/scaffolds in association with stem cells and growth factors to improve bone repair (Figure 1).

## 5. Mesenchymal Stem Cells

Mesenchymal stem cells (MSCs) were defined by Friedenstein et al. for the first time in 1970 as fibroblastic cell types that could produce clonal colonies with the capacity to generate bone and reticular tissue in guinea-pig models [57]. Thus, the International Society for Cellular Therapy (ISCT) defined criteria to identify human mesenchymal stem cells (hMSCs). Indeed, MSCs must be positive for CD73, CD90 and CD105 markers (>95%) and negative for specific antigens, such as CD45, CD34, CD14 or CD11b, CD79α or CD19, and HLA class II (<2%) [58]. hMSCs are plastic adherent cells that can differentiate into three cellular lines (osteoblasts, adipocytes, and chondrocytes) after exposure to certain soluble factors in the microenvironment [59,60]. Indeed, osteogenic differentiation typically involves the use of dexamethasone, β-glycerolphosphate, and ascorbic acid. Adipogenesis protocols also utilize dexamethasone, in addition to isobutylmethylxanthine and indomethacin [61]. Whereas, chondrogenesis protocols typically utilize dexamethasone, ascorbic acid, sodium pyruvate, TGF-β1 and a combination of insulin-transferrinselenium (ITS) [62]. Manfrini et al. [61] employed an isolation method that may be suitable for obtaining a cell population containing hMSCs (CD105, CD90, and CD73 positive in agreement with ISCT) from iliac crest bone marrow aspirates although contaminant cells from the hematopoietic lineage are found in the early cultivation stages as well as other authors. Indeed, this method is a common approach to obtain human mesenchymal stem cells.

Autologous cell transplantation is a good treatment option for large bone defects because it eliminates problems, such as limited autologous bone availability and allogenic bone immunogenicity. There are two main clinical application forms of cell therapies in bone regeneration: i) Cell therapies without expansion in culture and ii) cell therapies with ex vivo expansion [63]. In the first case, cells are harvested during an operation. For example, in 2010 Jäger et al. [64] successfully treated over 100 patients with local bone healing disorders using a biomaterial composite in association with bone marrow aspiration concentrate. Interestingly, their study has found that the use of bone marrow aspiration concentrate reduces the harvest of autogenous bone by 50% without negative effects on bone healing. 

On the other hand, the second clinical application form includes the autologous cell transplantation after ex-vivo cultivation. In this contest, Nöth et al. [65] reported that a mixed cell population from bone marrow cells, tissue repair cells (TRCs), was cultivated over 12 days under GMP conditions and then transplanted autologously with a tricalcium phosphate biomaterial (TCP) within the framework of core decompression.

The main drawbacks of MSCs cultivation regard the quality of the cell therapy treatment and the biological characteristics of MSCs.

Recently, Seebach and collaborators demonstrated in a first clinical phase-I trial that cell therapy with fresh autologous bone marrow mononuclear cells is safe and feasible, as well as probably efficacious when seeded onto β-TCP in situ in patients with proximal humeral fractures, thus a forthcoming clinical trial phase-II is needed [66].

Moreover, a study conducted by Sponer et al., combined autologous MSCs with a β-TCP biomaterial in human bone defects repair. Their data demonstrated that the addition of MSCs resulted in more trabecular remodeling in femoral defects [67,68].

In addition to bone marrow, hMSCs have also been found in many adult tissues, including the synovial membrane (SMSCs) [69], adipose tissue (ADSCs) [70], dental pulp tissue (DPSCs) [71] or perinatal tissue [72], such as umbilical cord blood (UCB) and umbilical cord tissue (UC).

For instance, Hatakeyama et al. reported that osteogenic and adipogenic differentiation of hMSCs derived from the knee bone is better than hMSCs derived from the hip bone [73].

Furthermore, osteogenesis and adipogenesis in hMSCs were studied in several regions of human umbilical cord. In particular, Mennan et al. demonstrated the best hMSC differentiation results in Wharton’s jelly region [74]. Zhang and his collaborators compared hMSCs derived from several tissues, including four from dental origins, to identify the best source of cells used in bone repair. Their results show that periodontal ligament stem cells (PDLSCs) are an optimal alternative to BM-MSCs [75].

However, in tissue engineering the most common sources of hMSCs from adult tissues remain bone marrow and adipose tissue. The choice of these two cell types is mainly due to the number of cells that can be harvested and the low risk associated with clinical practice procedure for obtaining these cells [62]. hMSCs play a key role in bone repair after fracture [76]. A study by Obermeyer et al. reports that MSCs, when isolated from bone marrow from transgenic Green Fluorescent Protein (GFP) C57BL/6 mice and administered intravenously following fracture can migrate to the defect site and contribute to fracture repair [77].

Recently, Hernigou el al. [78] compared the human bone marrow mesenchymal stromal cell behavior when grafted onto two different biomaterials: the cancellous devitalized Tutoplast^®^-processed bone (TPB) and the synthetic hydroxyapatite/β-tricalcium-phosphate (HA/βTCP). They showed that cell adhesion is two times favored on TPB in vitro and in vivo respect to HA/βTCP.

## 6. Biomaterials

Tissue engineering is an interesting field of study especially due to the increasing need for grafting materials. It is known that bone is composed of: (i) 50–74 wt% mineral phase (mainly HA 45–58%, carbonate ∼4%, citrate ∼0.9%, sodium ∼0.7%, magnesium ∼0.5%, and many other trace elements, such as F^−^, K^+^, Sr^2+^, Pb^2+^, Zn^2+^, Cu^2+^, Fe^2+^, (ii) 16–40 wt% organic (85–90% collagen) and (iii) 10 wt% water [11,79]. Thus, several researchers have tried to develop new biomaterials/scaffolds, to be used as substitutes inspired by bone composition and structure [80]. Furthermore, these biomaterials must have specific characteristics: (i) biocompatibility, or the ability of a material to perform without a host immune response, (ii) biodegradability, or the capacity of the biomaterial to decompose when new bone is formed, (iii) specific structure characteristics (e.g., porosity) and appropriate osteoinductive/osteoconductive properties to stimulate cellular proliferation and osteogenic differentiation in the healing site [81,82].

Moreover, in tissue engineering there are three main strategies for bone repair based on the severity of the trauma: (i) Direct biomaterial implantation, (ii) Stem cell isolation from patients and seeding on biomaterial as freshly harvested cells (e.g., bone marrow concentrate) or after expansion in vitro and (iii) cell harvesting, expansion in vitro and seeding on biomaterial using growth factors or other small molecules before implantation to the defect site. 

Herein, the most commonly used biomaterials in bone repair, such as ceramics and polymers, are reviewed in relation to composite scaffolds, which are a combination of both these biomaterials.

## 7. Ceramic Biomaterials

Ceramics have the advantage of being biocompatible with the human body while being resistant to compression and corrosion. However, these biomaterials have some disadvantages, such as brittleness and low strength [83]. Ceramics have many applications as biomaterials (e.g., on articulating surfaces) due to their chemical/physical properties. The most common ceramic biomaterials are composed of calcium phosphate (CaP) and tricalcium phosphate (TCP) [82]. In a pioneering study, Ishikawa et al. [4] compared three commercially ceramic-derived substitutes with different compositions: hydroxylapatite (HAp, Neobone^®^), carbonate apatite (CO_3_Ap, Cytrans^®^) and β-tricalcium phosphate (β-TCP, Cerasorb^®^). Their results demonstrated that CO_3_Ap shows limited dissolution and major stability under physiological conditions (pH 7.3) compared to other experimental groups.

It has been proven that cationic substitution (e.g., Sr^2+^ or Mg^2+^) in CaP-based biomaterials improve the mechanical properties and change the chemical/physical properties of CaP (e.g., crystallinity, microstructure, and solubility) [84]. In 2017, Montesi et al. characterized a strontium-doped HA cement (with different strontium concentrations) enriched with sodium alginate demonstrating in vitro that Sr^2+^ has the capacity to induce osteogenic differentiation [85,86]. In addition, Barbanti Brodano and his collaborators tested two other commercial hydroxylapatite-derived (HA-derived) biomaterials known as Sintlife (Mg^2+^-doped HA) and Engipore (high-porosity HA) in association with hMSCs derived from the bone marrow of adult orthopedic patients suffering from spine fusion in order to create a personalized approach to therapy for use in clinical practice. Their results suggest that Engipore biomaterials are better that Sintlife since the former induces cellular proliferation and focal adhesion kinase activation in hMSCs [7].

In another study, Sun and Yang [87] showed that the osteoinductivity of CaP-based biomaterials can be improved using recombinant human bone morphogenetic proteins (rhBMPs).

In recent years, there has been great interest in tissue engineering for the magnetic activation of biomaterials in order to carry specific growth factors to critical bone defects areas [88]. To this end, the characteristics of magnetic HA were studied by Panseri et al [89]. In vitro, this magnetic biomaterial induces cellular proliferation without the negative effects caused by magnetite. Subsequently, these results were confirmed by Russo et al. [90] who tested this biomaterial in a preclinical study using the rabbit model. Their results demonstrate that magnetic HA shows the analogous effects of commercial HA without the adverse effect attributable to magnetite.

## 8. Polymers

Polymer biomaterials can be natural and synthetic. Natural polymers mimic the structure and biochemical properties of the natural bone organic matrix; although, natural polymers have some disadvantages, such as poor thermal stability. Natural polymers include, for instance, collagen or chitosan. Collagen is the most abundant protein in the bone matrix [91]. Unfortunately, pure collagen have not good mechanical proprieties, thus it is usually associated, for instance, with ceramic biomaterials in bone tissue regeneration.

Chitosan is a linear polysaccharide that has amino groups on its surface that enhance interaction with glycosaminoglycans and proteoglycans, stimulating the cytokines and GFs that are important for tissue regeneration [92,93,94].

Another natural bone substitute also used in tissue engineering is the demineralized bone matrix. This substitute derived from bones lacking mineral components while being rich in type I collagen and other growth factors [95,96,97,98]. Recently, Desai et al. demonstrated that using demineralized bone matrices in association with bone marrow concentrate can increase the success of non-union treatment [99,100].

Among the most important synthetic polymers to be found are the following: poly (ε-caprolactone) (PCL), polylactic acid (PLA), polyglycolide (PGA) and the copolymer of poly-(DL-lactic-co-glycolic-acid) (PLGA). PCL is an aliphatic polyester biomaterial, which was approved by the FDA since it is multiform and highly biocompatible [101]. PLA and PGA are unsuitable as biomaterials for bone tissue regrowth because of the low osteoconductivity and compressive strength. PLGA copolymers with several ratios of PLA and PGA are more soluble and have major osteoconductivity, whereas the degradation time of the latter can be controlled [102,103]. However, the use of synthetic polymers has some disadvantages due to their degradation. This process gives rise to acid products that can alter the local "microenvironment" causing local change in pH [91]. These polymeric biomaterials are employed as drug-delivery systems for bone tumor treatment. This strategy improves the local administration of antitumor drugs. Indeed, several studies demonstrated that analyzed drug-delivery systems, such as chitosan/paclitaxel [104], gelatin/doxorubicin [105], or PLGA-based hydrogel biomaterial favor the treatment of osteosarcoma [54].

## 9. Composite Biomaterials

Composite biomaterials derive from a combination of polymers and ceramics scaffolds. This type of biomaterial has certain characteristics such as high biocompatibility, mechanical hardness, and load-bearing capabilities that make these biomaterials suitable in tissue engineering [106].

Some recent in vitro and in vivo studies analyzed composite biomaterial formed by porous HA and collagen to evaluate the biological and mechanical effects of scaffolds. Specifically, Mazzoni et al. [10] evaluated the in vitro biocompatibility, osteoconductivity, and osteoinductivity properties of scaffolds composed of HA (Pro Osteon 200) and microfibrillar collagen (Avitene) that are also known as Coll/Pro Osteon200, using hMSCs derived from human adipose tissue. The results show that this biomaterial can induce osteogenic differentiation in hMSCs because induce the up-regulation of osteogenic genes with an increase of cell viability and matrix mineralization without toxic effects. D’Agostino et al. employed the same biomaterial in maxillofacial surgery showing that it is an optimal scaffold for zygomatic augmentation surgery [107]. In the same period, Calabrese et al. analyzed both the in vivo and ex vivo characteristics of cell-free collagen-HA scaffolds [108]. Subsequently, they evaluated implanting collagen-hydroxyapatite scaffold in association with human adipose-derived mesenchymal stem cells to determine if bone formation could be influenced by human stem cells [109], concluding that adding human stem cells can improve the bone repair process.

Other studies have investigated new techniques to improve the functional performance of biomaterials. For instance, a study conducted by Wang et al. suggested that composite scaffolds composed of a PLLA / β-TCP matrix grafted with gelatin/hydroxyapatite represent a good candidate for bone repair [110]. Moreover, Arafat et al. analyzed the proprieties of a scaffold composed of poly (ε-caprolactone)/tricalcium phosphate (PCL/TCP) with carbonated hydroxyapatite (CHA)-gelatin composite. This study indicated a strong increase in cellular proliferation and differentiation of BMSCs grown on this scaffold [111]. Finally, several composite biomaterials have been employed as drug delivery-systems, such as COLL/HA/cisplatin-derived scaffolds [112,113].

All these results suggest that composite biomaterials are excellent alternatives in tissue engineering.

## 10. Growth Factors and Platelet-Rich Plasma (PRP)

Generally, the three key elements in tissue engineering fields are stem cells, biomaterials and growth factors (GFs) [114]. In particular, GFs play an important role in tissue regeneration and they are involved in new clinical strategies to improve the bone healing process [115]. In the human body, GFs are produced from bone marrow stromal cells, endothelial cells, fibroblasts, inflammatory cells, and osteoblasts during the bone repair process [116]. In this review, the major GFs involved in the bone remodeling cascade were considered: bone morphogenetic proteins (BMPs), platelet-derived growth factor (PDGF), vascular endothelial growth factor (VEGF), fibroblast growth factor (FGF), transforming growth factor-β1 (TGF-β1), and insulin-like growth factor 1 (IGF-1).

GFs include BMPs that belong to the transforming growth factor (TGF)-β superfamily [117]. To date, twenty BMPs have been identified, including BMP-2/-4/-5/-6/-7 [118] as being involved in osteogenic differentiation. A recent study showed that BMP-2, in association with particular scaffolds composed of hydrogel enriched with alginate, are able to induce BM-MSCs cell line to differentiate osteogenically [119]. Many researchers have invested efforts in producing recombinant GFs for use in therapeutic strategies. Only a few recombinant GFs were approved due to problems related to cost, safety or limited half-life. Currently, recombinant BMP-2 (rhBMP-2) is commercialized as INFUSE^®^ or InductOS^®^ in US and Europe, respectively [120]. rhBMP-2 is a lyophilized product (with absorbable collagen sponge as a carrier) used for the treatment of spinal fusions, fractures, bone defects and, since 2007, in maxillofacial surgery [121]. However, there are some questions related to the large doses of BMPs utilized in the treatment of spinal fusion and the risk of cancer onset after surgery [122].

Subsequently, the FDA approved the recombinant BMP-7 (rhBMP-7) protein, introduced to the market as a osteogenic protein-1 (OP-1), that contains rhBMP-7, type I bovine collagen matrix and the putty additive carboxymethyl cellulose sodium (CMC) [123,124,125]. Like BMPs, platelet derived growth factor (PDGF) also plays an important role in bone healing since it is secreted by platelets at the site of fracture during early tissue repair [126]. Among multiple isoforms, PDGF-BB is the growth factor in this family that can bind to all isoforms of the PDGF receptor [127].

Several studies reported that the association between PDGF and biomaterials is effective in bone regeneration [128,129]. Raghavendran et al. showed that PDGF-BB acts synergistically with biomaterials, such as PLLA/Col/HA and PLLA/HA to enhance osteogenic differentiation potential. Therefore, this combination can be used for bone tissue regeneration. Paglia et al. conducted an in vivo study using a rabbit model to determine the effects of PDGF-BB in association with thiol-modified hyaluronic acid (TMHA) hydrogel on intervertebral disk degeneration. Their results indicate that PDGF-BB decreases disc degeneration and can prevents both apoptosis and matrix production when delivered in a TMHA gel biomaterial. Di Giovanni et al. reported that recombinant human platelet-derived growth factor-BB and beta-tricalcium phosphate (rhPDGF-BB/β-TCP), which was approved by the FDA and commercialized as AUGMENT^®^ Bone Graft in 2005 for alveolar bone regeneration [130].

As noted previously, bone remodeling is a process that involves the removal of mineralized bone followed by the formation of bone matrix by osteoclasts and osteoblasts, respectively [22].

Transforming growth factor beta 1 (TGF-β1) is a member of the transforming growth factor (TGF)-β super family [131] involved in bone remodeling with insulin-like growth factor 1 (IGF-1). TGF-β1 with other members of the TGF-β super family, such as myostatin and activin A, can modulate osteoclastogenesis since it can act on mechanisms that depend on RANKL-RANK interplay [131]. Another function of TGF-β1 is to recruit BM-MSCs to remodeling sites: it is a chemotactic agent towards BM-MSCs, while not being responsible for osteoblastic differentiation. Thus, this role is reserved to IGF-1, which can induce recruited BM-MSCs to differentiate in osteoblasts [82,132]. Gugjoo et al. have shown that treatment with BM-MSCs combined with IGF-1/TGF-β1 in laminin gel scaffolds can improve osteochondral defect repair in rabbit models [133].

In the first phase of secondary fracture healing, growth factors are required to re-vascularize the damaged sites and to induce new bone formation [134]. In this contest, when a fracture interrupts the blood supply, vascular endothelial growth factor (VEGF) is required to repair the damaged site [82]. The VEGF family is composed of VEGF-A (VEGF), VEGF-B, PlGF, VEGF-C, and VEGF-D [135]. VEGF is its most abundant form and is fundamental to the proliferation, migration and activation of endothelial cells. Moreover, it plays an important role in the promotion of permeability and fenestration of blood vessels [136]. VEGF expression is regulated by several GFs involved in bone repair, such as members of the TGF-β superfamily, in particular TGF-β1, TGF-β2, BMP2, BMP4, and BMP7 [137,138,139], insulin-like growth factor (IGF) [140] and FGF-2 [141]. FGF is another GF implicated in the formation of new blood vessels. Specifically, FGF-2 seems to up-regulate VEGF expression [142]. All these data suggest that the modulation of VEGF levels in osteoblasts can provide a basis for strategies to control bone repair and regeneration. Several abovementioned GFs, such as TGF-β1, PDGF-BB, VEGF-A and IGF-I, are present in Platelet-rich plasma (PRP). PRP is a concentrate of activated platelets in a small volume of plasma. The platelets are obtained from whole blood by differential centrifugation and are activated with a mixture of thrombin and calcium [143,144]. Upon activation, exocytosis of the dense granules and alpha granules (containing the growths factors) takes part. The α–granule contents must be released from their intracellular repository in order to achieve their physiologic function [145].

In literature, there are conflicting results [146,147] about the use of PRP in bone regeneration. Bianco et al. have suggested that PRP seems to be more effective than single recombinant GFs due to the synergism among all GFs [148]. In a recent work, Shafieian et al. studied the potential therapeutic of PRP in association with ADSCs seeded on HA/TCP biomaterial to evaluate the healing response of canine alveolar surgical bone defects. The results show that ADSCs seeded on HA/TCP in combination with PRP can promote bone repair more effectively than control groups represented by those defects treated only with PRP and HA/TCP [149].

Conversely, in two different studies, Mooren et al. [150,151] reported no detectable benefit from the combination of PRP and autogenous grafts in goat critical size frontal bone defects. Ranly et al. showed that PRP added to demineralized bone matrix decreased its osteoinductivity in a nude mouse model [152]. 

Thus, more investigations are needed on PRP and its role in the osteogenic process. 

## 11. Conclusions

Large bone defect repair is a serious problem that requires specific and costly management. For this reason, there is a need to identify novel therapeutic strategies, which will lead to improved patient outcomes. To this purpose, tissue-engineering employs a combination of stem cells, biomaterials/scaffolds and bioactive agents to repair damaged bone and to improve bone regrowth. hMSCs can be obtained from several prenatal and adult tissues. Future works should be directed to a better understanding of the different aspects of these cells. As we know, tissue engineering is providing for the development of various biomaterials that can induce and support bone regrowth after damage. Currently, there are several biomaterials that can be employed in clinical practice. The most common are ceramic, polymeric, and composite biomaterials that mimic bone structure and composition. The numerous characteristics of these scaffolds make them suitable for use in tissue regeneration. However, other studies are required to limit possible side effects and improve their ability in drug-delivery or rapid bone regeneration with specific antitumor drugs and growth factors, respectively. In this contest, the choose of a suitable biomaterial as carrier system is important to allow the localized and sustained release of single or multiple GFs such as BMPs, PDGF, VEGF, FGF, TGF-β, and IGF-1 characterized by shorter half-life and rapid degradation. PRP seems to be safe with good beneficial therapeutic effects. In literature, there are also conflicting results about the effectiveness of PRP in relation to rhGFs (i.e., rhBMP-2 or rhBMP-7) use. For these reasons, new studies are needed to define an optimal method of preparation and administration for PRP and to identify the most effective system for improving the bone healing process.
